# Bitwise efficiency in chaotic models

**DOI:** 10.1098/rspa.2017.0144

**Published:** 2017-09-06

**Authors:** Stephen Jeffress, Peter Düben, Tim Palmer

**Affiliations:** Department of Atmospheric Physics, University of Oxford, Oxford, UK

**Keywords:** information, chaos, supercomputing, inexactness, precision, field programmable gate array

## Abstract

Motivated by the increasing energy consumption of supercomputing for weather and climate simulations, we introduce a framework for investigating the bit-level information efficiency of chaotic models. In comparison with previous explorations of inexactness in climate modelling, the proposed and tested information metric has three specific advantages: (i) it requires only a single high-precision time series; (ii) information does not grow indefinitely for decreasing time step; and (iii) information is more sensitive to the dynamics and uncertainties of the model rather than to the implementation details. We demonstrate the notion of bit-level information efficiency in two of Edward Lorenz’s prototypical chaotic models: Lorenz 1963 (L63) and Lorenz 1996 (L96). Although L63 is typically integrated in 64-bit ‘double’ floating point precision, we show that only 16 bits have significant information content, given an initial condition uncertainty of approximately 1% of the size of the attractor. This result is sensitive to the size of the uncertainty but not to the time step of the model. We then apply the metric to the L96 model and find that a 16-bit scaled integer model would suffice given the uncertainty of the unresolved sub-grid-scale dynamics. We then show that, by dedicating computational resources to spatial resolution rather than numeric precision in a field programmable gate array (FPGA), we see up to 28.6% improvement in forecast accuracy, an approximately fivefold reduction in the number of logical computing elements required and an approximately 10-fold reduction in energy consumed by the FPGA, for the L96 model.

## 1. Introduction

The power wall that traditional supercomputing is approaching means that future improvements in weather and climate forecasting may require a substantial increase in energy consumption. For example, it has been estimated that running a global cloud-resolving climate simulation for a century would need the type of ‘exascale’ supercomputing that is projected to consume gigawatts of power if computing technology continues to scale as it currently does [1,2]. The present study contributes to the effort to reduce energy consumption and improve forecast accuracy in climate modelling by the use of inexact computing strategies. Below we explain how inexact computing works, why it is beneficial for climate modelling, and why the development of a bitwise information metric is an important contribution.

### (a) Inexactness in computers

Computers use energy to achieve exact calculations in two ways: (i) by using high voltages to ensure low bit flip error rates and (ii) by using more bits to increase precision in variables. At the circuit level, a computer requires energy to change the voltage of a transistor from low to high. We refer to this as ‘flipping’ a bit between zero and one. The low voltage is usually 0 V and the high voltage is often 5 V. Using high voltages for the upper limit requires more energy but results in less chance of a bit flip error. Using lower voltages to conserve energy while accepting errors is known as ‘voltage overscaling’. It is often difficult to isolate the parts of a computer that can tolerate occasional bit flip errors from the parts that cannot. However, if technologies and algorithms are designed to gracefully combine traditional processors with voltage overscaled chips, then large gains in energy efficiency may be possible. For example, Palem [3] found a fourfold improvement in energy efficiency if a 1% bit flip error rate could be tolerated.

In addition to using high voltages, the other way that computers ensure high levels of exactness is by representing variables with a large quantity of bits and more advanced number representation formats. In most computer languages, the simplest format is an 8-bit integer and the most advanced is a 64-bit floating point. The decision to use fewer bits or to switch from a floating point to an integer is called ‘reduced precision’. Reducing precision will typically require more engineering development time and produce less exact calculations, but will achieve greater energy efficiency.

In both the integer and floating point number formats, the amount of bits used specifies the quantity of unique numbers that can be represented. For example, 16 bits provides 2^16^=65 536 unique numbers, while 64 bits provides 2^64^=18 446 744 073 709 551 616 unique numbers, and this is true regardless of whether it is an integer or a floating point. The difference in the formats is in the upper/lower bounds and the size of the increments between each representable number. The standard integer format represents all the integers between a maximum and minimum determined by the number of bits. A more general version of the integer format, called ‘scaled integer’ or ‘fixed point’, contains a scaling factor and offset that sets the upper/lower bounds and fixes a constant increment (thus allowing decimal number representation). Appropriately choosing the scale and offset, and then possibly redesigning algorithms to fit these choices, is the primary reason that switching from a floating point to a scaled integer requires longer engineering development time.

The floating point format with 32 or 64 bits (defined in IEEE standard 754 [4]) is convenient from the code writer’s perspective because of its fixed upper/lower bounds and non-uniform distribution of numbers. Having spacious gaps among large numbers and tiny increments near zero provides variables with both a large upper/lower range and high precision where it is usually needed the most. However, the simplicity that the non-uniform distribution provides for the programmer is traded off in the complexity of the hardware needed for the arithmetic at the circuit level.

As is illustrated in §4 of this paper, floating point arithmetic circuits occupy a larger chip area, require more clock cycles and consume significantly more energy than integer arithmetic circuits. Details of how much energy can be saved by switching to scaled integer formats depend heavily on the distribution of arithmetic operations in an algorithm. But it is generally understood that great energy savings are possible if the investment in engineering time can be made. In a recent example [5], a 16-bit integer version of a deep learning algorithm was created on a dedicated hardware platform and a 10-fold gain was found in energy efficiency compared with the 32-bit floating point version on a standard processor. We end up finding a similar performance gain in this study.

### (b) Inexactness in climate models

Although a natural assumption is that inexactness in calculation translates to inaccuracy in climate modelling, the careful inclusion of random errors and imprecision can often be beneficial. The two main reasons that this can happen are that (i) adding small amounts of randomness provides a better representation of small-scale chaotic processes and (ii) reducing precision in selected variables allows computational resources to be reinvested for better use.

The inclusion of specific kinds of randomness into climate simulations is known as ‘stochastic parametrization’. The physical basis for stochastic parametrization is related to the phenomenon that is known as the ‘butterfly effect’ and whose difficult solution is known mathematically as the ‘closure problem’ in multi-dimensional systems. Chaotic processes are often too small to be resolved in models, but they have important impacts that cannot be represented by a deterministic function of large-scale variables [6]. In the last decade, studies have consistently shown the benefits of using partially random instead of fully deterministic techniques to represent the unresolved processes, and today stochastic parametrization is routinely used in operational weather forecast centres [7].

The second argument for achieving greater accuracy through inexactness is that reducing precision allows computational resources to be put to better use. Although it is standard practice in scientific computing to use 64-bit double precision floating point numbers (for reasons discussed above), it is almost certainly a poor allocation of computational resources for achieving maximum efficiency in climate simulations. For example, Düben *et al.* [8] and Düben & Palmer [9] study the effect of reducing precision on the long-term climatology of Intermediate General Circulation Model (IGCM) simulations and on the short-term weather forecasts in the Open Integrated Forecasting System (OpenIFS) operated by the European Centre for Medium-Range Weather Forecasts (ECMWF). It was found that 98% of the 64-bit IGCM calculations could be performed with only 15 bits, without degradation in simulation quality. In OpenIFS, the relatively simple conversion of variables from a 64-bit double to a 32-bit single floating point (simple compared with scaled integer conversion) achieves the same forecast accuracy with half the bits and up to 40% less computation time.

The reason that computational savings are relatively quickly realized when switching from double to single floating point formats is because redesigning algorithms is typically not required, and separate arithmetic circuitry for these two formats is standard on modern microprocessors. By contrast, converting to scaled integer or low bit floating point formats will probably require custom hardware for the large efficiencies to manifest. For example, it is not common to have 16-bit ‘half-precision’ arithmetic units in a microprocessor (hopefully more common in the near future), so a compiler will probably up-convert half-precision floats to single precision floats to perform arithmetic. In this situation using half-precision may still save computational resources in storage and data transport, but not likely in computation.

To take full advantage of the efficiency that reduced precision provides, custom hardware is often built for the application. For example, even though graphic processing units (GPUs) are now used for many purposes, they were originally designed and built specifically for rendering graphics in video games. The most common way to begin designing hardware specific to applications is to use a field programmable A (FPGA). Because FPGAs are coded in the same hardware description language (e.g. Verilog) as custom printed chips, the reconfigurable FPGAs are typically used to test circuit designs and work through bugs on the logic level before printing onto fixed hardware. In recent years, however, FPGAs have been used not as a prototype, but as production-level accelerators for scientific and financial computing. This transition is largely due to new technologies such as Maxeler Technologies’ FPGA-based data flow engines. These devices streamline the process of designing massively parallel arithmetic structures at the hardware level for complex algorithms. The technology is beginning to show promise for simplified atmospheric model simulation. For example, Oriato *et al.* [10] encode the dynamical core of a limited area meteorological model on an FPGA and report a 74× speed-up compared with a 12-core multi-threaded central processing unit (CPU) implementation. A related study [11] uses the technology to integrate a global atmospheric shallow-water system to achieve a 14× acceleration and a 9× increase in energy efficiency compared with a hybrid CPU–GPU implementation. In both of these studies, a variety of reduced precision techniques are used to maximize the efficiency of the FPGA’s finite computational resources.

### (c) Bitwise information content

A common theme emerging from the recent work on inexact computing for climate models is the need to efficiently and robustly determine which bits in the model contribute most significantly to simulation or forecast quality. It is for this purpose that we introduce the bitwise information metric in this paper. Below we explain why the need for efficiency and robustness has arisen and briefly outline how this study will unfold.

In the reduced precision climate modelling studies mentioned in the previous section, the precision analysis is typically accomplished by a guess-and-check approach; in other words, by running a control model, then removing or randomizing certain bits and then rerunning a reduced precision version. This process not only consumes large amounts of time, but also can require significant computational energy of its own for large models. The bitwise information metric presented in this paper requires only one high-precision run to determine the value of each of the bits in the simulation.

The other theme emerging in previous studies is the need for the precision analysis to be less sensitive to a model’s time step. Ideally, we would like a precision analysis to be more reflective of the dynamics and uncertainty in the chaotic system, rather than the size of the time step used in the numerical integration. It is often the case that a small time step must be used to maintain the numerical stability of a model integration—and a precision analysis performed on the model’s output will certainly be influenced by the choice of time step—but it is not desireable to have a precision analysis that tends to infinite precision as the time step tends to zero. The bitwise information metric introduced in this study is not significantly influenced by decreasing a time step beyond that which is needed for numerical stability. However, it is highly influenced by the size of the uncertainty and chaos in the model. Thus, the metric presented quantifies information in a manner that is more a property of the underlying dynamical system and less a property of the numerical method.

The remainder of this study is organized as follows. Section 2 introduces our bitwise information metric by providing the mathematical basis and relationships to other forms of information theory used in climate science. Section 3 applies the information metric to the Lorenz 1963 (L63) model. The result is a quantification of the information contained in each bit of a 32-bit L63 integration. Section 4 applies the information metric to the Lorenz 1996 (L96) model, and then uses the results of the analysis to guide the design of reduced-precision FPGA implementations. The reduced-precision FPGA implementations of the L96 model show the trade-off between numeric precision, spatial resolution, forecast accuracy and energy consumption. The main findings are summarized in the conclusion.

## 2. Mathematical framework

In this section, we define the bitwise information metric. Our approach is similar to other usages of information theory in climate science such as predictive information, relative entropy and mutual information [12–14]. However, these usages are typically designed to relate climate events to one another for statistical significance or predictive purposes, whereas our goal is to relate chaotic predictability to the act of bit flipping in a simulation. Thus, instead of defining an entropy to be conditioned on potentially related climate events, our measure of entropy is conditioned on the true–false value of the bits used in the number representation format of the variables in the model. Information is then the contribution that bit flipping makes towards improving forecasts, given that natural uncertainties exist and grow chaotically in the system. The natural uncertainties, such as the initial condition or stochastic parametrizations, are analogous to noise in the communication channel of Shannon’s 1948 information theory [16]. The accuracy of forecasts is measured by reducing the entropy of a forecast probability distribution function (PDF) with bin widths the size of the natural uncertainty. Thus, a bit has high information if its flipping results in a statistically significant improvement (reduction in entropy) in the forecast PDF at a given forecast time. As the forecast time extends, the information content of all bits will eventually become negligible because the natural uncertainty growing in the chaos eventually removes all predictability. Integrating over all useful forecast periods therefore provides a robust quantification bitwise information content that does not grow indefinitely for decreasing time step.

### (a) Method requirements

Let ***x***(*t*)={*x*(*t*),*y*(*t*),*z*(*t*),…}^T^ be the state vector of a multi-dimensional, real-valued chaotic system (*d*/*dt*)***x***(*t*)=*f*(***x***), and assume that we have access to a time-series sample of one element, namely *x*(*t*). Often the sample time series will come from the numerical integration of *f*(***x***), as it will in later sections of this paper, but the metric presented here could also be applied to a time series obtained from observational measurement of a chaotic system. The simplest form of the metric is applied to a single scalar element *x*(*t*), but a more advanced version (discussed at the end of the section) can include the full vector *x*(*t*).

The method applied to the scalar *x*(*t*) is performed as follows. Assume that we have *N* time samples *x*(*n*Δ*t*), *n*=1,2,…,*N*, separated by time step Δ*t*. This time step needs to be small compared with the time scales of variability in the system, such as the time step required for accurate numerical integration, but the method is not sensitive to ever-decreasing values of Δ*t*, as is explained in the text below equation (2.4).

The next requirement is that the system being modelled should have a natural uncertainty of size *h* when projected onto *x*(*t*). The uncertainty could arise from various (or a combination of) sources including initial condition uncertainty, measurement error or the stochastic term added to each time step (e.g. for stochastic parametrization or for reduced dimensional models where noise is introduced to represent the missing dimensions [15]). Similar to the time step requirement, the uncertainty *h* needs to be small compared with the variability of *x*(*t*). In contrast to the time step, the information metric is highly sensitive to the value of *h*. This sensitivity is intentional as the goal of the metric is to relate the information in the bits to the growth of the chaotic uncertainty.

Another important matter is the size of the bin widths used for the PDFs of *x*, because it determines the length of the data sample required. We have found it best to set the bin size to the size of the uncertainty *h*. This is a natural choice for PDF bin widths because the uncertainty gives us no reason to distinguish among values inside the bin. The bin size sets the length of the time series required to achieve a stable PDF for the following reason: if we let *h* tend to zero, it would not be clear when (if ever) the PDF would converge; because the system is chaotic and the phase space of *x* is probably fractal. Also, due to the fractal nature of the system, inside every phase space increment in *x* we will have additional structure which requires additional precision to resolve. Thus in order for our precision analysis to be well defined, we require our time series sample *x*(*n*Δ*t*) to be sufficiently long to produce a stable PDF with bin width *h*.

Although it would be highly unlikely to be otherwise, it is also important that the same numeric type, i.e. the protocol that maps bit positions to real numbers, must be used throughout the time series. This means that each time sample *x*(*n*Δ*t*) is stored using *B* bits of precision (e.g. *B*=64 bits for a double precision floating point). To denote each individual bit, let *x*_*b*_(*n*Δ*t*) be the value of the bit in position *b*=1,2,…,*B* at time *n*Δ*t*. Note that *x*_*b*_(*n*Δ*t*) is always either zero or one, which allows conditional PDFs to be constructed in a very natural way, i.e. *p*(*x* | *x*_*b*_=0,1).

### (b) Information content definition

We now define the information content in each bit of the system following [16]. The information content of the bit in position *b*=1,2,…,*B* comes from the difference in entropy that arises from whether the bit is a one or zero. In order to find this difference, we first define the *unconditional* entropy *H*_*x*_ as

2.1 $$\begin{array}{r} H_{x}=-\sum_{i=0}^{M}p{\left(x\right)}_{i}\;\log\;p{\left(x\right)}_{i}. \end{array}$$

Here, *p*_*i*_(*x*) is the *i*th bin of the *M*-bin PDF of *x*(*t*). The size of the bin width in this PDF is the natural uncertainty *h* described above.

The *conditional* entropy *H*[*x*|*x*_*b*_(*τ*)=0,1] is then the entropy of *x* conditioned on whether the bit *x*_*b*_(*τ*), the bit in position *b*, is a zero or one at forecast time *τ*,

2.2 $$\begin{array}{rl} H_{b0}(\tau ) & =-\sum_{i=0}^{M}p_{i}[x|x_{b}(t-\tau )=0]\;\log\;\{p_{i}[x|x_{b}(t-\tau )=0]\}\\ \;\text{and}\;\;H_{b1}(\tau ) & =-\sum_{i=0}^{M}p_{i}[x|x_{b}(t-\tau )=1]\;\log\;\{p_{i}[x|x_{b}(t-\tau )=1]\}. \end{array}\}$$

Here, [*x*|*x*_*b*_(*t*−*τ*)=0,1] means the value of *x*(*t*), given that bit *x*_*b*_ was a zero or one at time offset *τ* in the recent past. For *τ*=0, the PDF is conditioned on the simultaneous value of the bit. In this case, it simply shows the bit’s function in the number representation. For example, the PDF conditioned on the sign bit at *τ*=0 would simply select the positive or negative regions of the unconditional PDF. As *τ* grows large, the conditional PDF becomes the same as the unconditional PDF because the chaos has removed the predictability for all bits. The information content *I*_*b*_(*τ*) of this bit and forecast time is, therefore, the difference between the unconditional and conditional entropies weighted by *q*_0_ and *q*_1_, the *a priori* likelihoods of the bit being a zero or one throughout the time series:

2.3 $$I_{b}(\tau )=H_{x}-q_{0}H_{b0}(\tau )-q_{1}H_{b1}(\tau ).$$

The total bitwise information content *J*_*b*_ is then obtained by integrating *I*_*b*_(*τ*) over all forecast times

2.4 $$J_{b}=\int_{\tau =0}^{\infty }I_{b}(\tau )\;d\tau .$$

This integral converges under the assumption of a finite uncertainty in the chaotic system. This uncertainty will eventually grow to remove all predictability in the system, meaning that there is a *τ* at which *I*_*b*_(*τ*) becomes and remains zero. This indicates that the integration of *I*_*b*_(*τ*) over all *τ* will be finite.

The fact that (2.4) integrates over all useful forecast times makes *J*_*b*_ insensitive to an ever-decreasing time-step size. Here, we assume that the error associated with the choice of numerical method and time step is small compared with the error associated with the growth of the uncertainty. In this case, as Δ*t* becomes very small, more precision is required to represent the change in the state variables over the small time step, so we would expect to see *I*_*b*_(*τ*) contain more information for more bits at very small *τ*. However, *J*_*b*_ is not heavily affected by this because *J*_*b*_ integrates *I*_*b*_(*τ*) over the useful forecast period, which is dominated by the growth of the uncertainty in the system. Thus, a smaller Δ*t* will better approximate integrals in *J*_*b*_, but decreasing Δ*t* beyond a certain point will have diminishing effects.

In contrast to the insensitivity to small Δ*t*, equation (2.4) is highly sensitive to the size of the uncertainty *h*. The reason for this is because the system is chaotic with exponential growth of uncertainty. This means that, for large *h*, forecast times will be short and the information in *J*_*b*_ will be small. For small *h*, the forecast times become long, which makes more bits contain more information. As *h* tends to zero, meaning no uncertainty, the information in all bits becomes very large. For this reason, we argue that the information quantified by *J*_*b*_ is more a property of the chaos and uncertainty in the model, and is less sensitive to the size of the model time step Δ*t* (and presumably less sensitive also to the details of numerical methods in other ways).

### (c) Multi-dimensional version

The method discussed to this point has been for the case that we possess only one element *x*(*t*) of the multi-dimensional chaotic system ***x***(*t*). If we have an additional element *y*(*t*), we can add the information that the bits of *x* hold for predicting *y*. To do this, we would add the following to (2.3):

2.5 $$+H_{y}-\{q_{0}H[y|x_{b}(\tau )=0]+q_{1}H[y|x_{b}(\tau )=1]\}.$$

This process can continue for each additional element of the system for which *x* may hold prediction power. Each addition would modify *I*_*b*_ and (2.4) may provide a better overall quantification of the information in bit *x*_*b*_. However, in practice, we expect that if bit *b* of *x* is important for predicting *y*, it is probably also important for predicting itself, so it probably already has a non-negligible information content. Thus, we would expect to see diminishing returns of adding more multi-dimensional elements into the analysis.

## 3. Bitwise information in Lorenz 1963

In this section, we apply our bitwise information analysis to the prototypical chaotic attractor known as the Lorenz 1963 (L63) model [17]:

3.1 $$\begin{array}{rl} \frac{dx}{dt} & =\sigma (y-x),\\ \frac{dy}{dt} & =x(\rho -z)-y\\ \;\text{and}\;\;\frac{dz}{dt} & =xy-\beta z. \end{array}\}$$

The model is a system of three ordinary differential equations originally obtained by simplifying the equations of turbulence arising in a thin layer of fluid when heated from below. Today the model is used less for its insights into fluid convection and more for its ability to illustrate general properties of chaos.

### (a) L63 model set-up

To obtain the sample time series required by our method, we integrate (3.1) numerically using the MATLAB development environment. After discarding the transient behaviour associated with a random initial condition, the model is integrated for 10^8^ model time units (mtu) with a fourth-order Runge–Kutta (RK4) method, and a time step of Δ*t*=0.01 mtu. The model parameters are set to the values most commonly used in studies of the system, *σ*=10, $\beta =\frac{8}{3}$ and *ρ*=28.

We assume an uncertainty of *h*=0.3 in all three elements of ***x***=[*x*,*y*,*z*]^T^ at all times in the integration. This uncertainty is interpreted as a measurement or initial condition error and is used as the bin width in PDFs of *x* as discussed in §2a. We also use the uncertainty to start a perturbation time series ***x***′(*t*). This perturbation is restarted every 30 mtu from the current value of ***x***(*t*) plus a random offset (distributed normally with mean zero and standard deviation equal to *h*). The perturbation time series is not necessary for the information analysis, but allows us to check for consistency when precision is reduced after the analysis (§3c).

All variables in the numeric integration are represented using the IEEE-754 32-bit floating point standard with a slight modification. The modification simplifies the information quantification because it greatly reduces the amount of redundant bit flipping when the exponent of *x* switches between positive and negative. The IEEE-754 standard for a single precision floating point is

3.2 $$\mathrm{real}\;\mathrm{number}={\left(-1\right)}^{b_{31}}\times 2^{\left(e-127\right)}\times \left(1+\sum_{i=1}^{23}b_{23-i}2^{-i}\right).$$

Our modified version is similar in that the sign bit *b*31 and fraction bits *b*0:*b*22 are the same as in the standard above. However, in the standard the exponent e is an *unsigned* integer made from bits *b*23:*b*30. This ‘unsigned’ property is the reason for subtracting 127 in the standard format (otherwise a negative exponent could not be achieved). Our slight modification makes the exponent a *signed* integer such that the first exponent bit gives the sign of the exponent and the last seven exponent bits give its value. With this change, when the exponent of *x* switches from positive to negative, only one exponent bit has to flip, instead of nearly all of the exponent bits when the IEEE standard is used.

### (b) L63 bitwise information results

**Figure 1.**
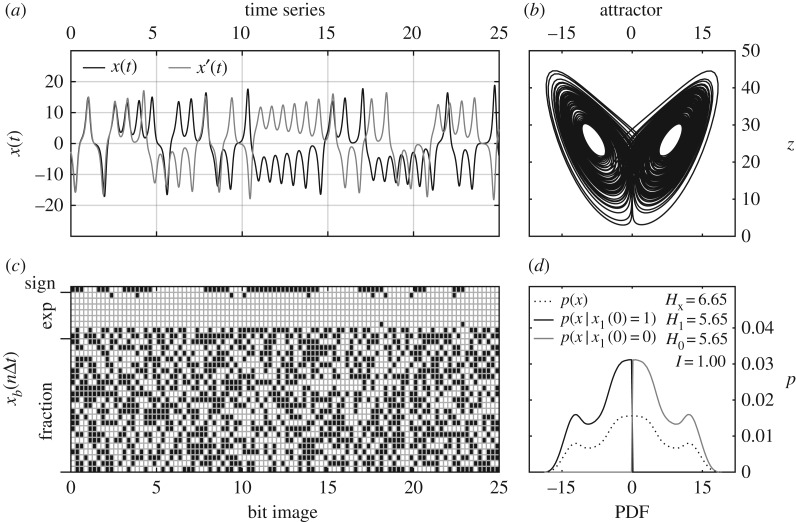
Time series, attractor, bit image and example information calculation from bit-conditional PDFs of the *x*(*t*) in the Lorenz [17] model with initial condition uncertainty *h*=0.3. (*a*) A 25 time-unit sample of *x*(*t*) (black line) from the Lorenz attractor (*b*) together with perturbation *x*′(*t*) (grey line) resulting from a slight difference in the initial condition. (*c*) The floating point bits *x*_*b*_(*n*Δ*t*) that hold the numeric value of *x* at several time samples. (*d*) The information content of the sign bit for predicting the current state of *x*, *I*=1.00, is obtained from the difference in entropy *H* between the unconditional PDF *p*(*x*) and the weighted average of the PDFs conditioned on the true–false value of the sign bit *p*(*x*|*x*_*b*=1_(*τ*=0)=1,0).

Figure 1 depicts how the bitwise information analysis is applied. Figure 1*a* shows a 25 mtu segment of *x*(*t*) and the perturbation *x*′(*t*). The bits comprising the value of *x*(*t*) at selected points in the segment are aligned in the time series in the bit image in figure 1*c*. In the bit image, we can see the sign bit flipping when *x*(*t*) goes from positive to negative, and the first bit of the exponent flipping when the magnitude of *x* changes between less than one and greater than one. Many of the exponent bits do not flip at all throughout the time series. This intuitively suggests that these bits do not have any information content. And this is found to be the case. In contrast to the exponent bits, fraction bits flip quite often and seemingly randomly. It is not immediately clear whether all the bit flips in the fraction contribute meaningful (predictive) information until the analysis is performed.

The PDFs in figure 1*d* illustrate how the unconditional entropy *H*_*x*_ (dotted line) and sign bit conditional *H*_1_, *H*_0_ (black, grey lines) give the information content *I* of the sign bit. The bin width used for the PDFs is the initial condition uncertainty *h*=0.3 as explained above. As expected, the conditional PDFs simply map out the positive and negative sides of the unconditional PDF. Following equations (2.1)–(2.3), the unconditional entropy at *τ*=0 is *H*=6.65, whereas the entropy conditioned on the sign bit has a weight average of *H*=5.65. The difference in entropy gives *I*=1 for the information content of the sign bit at zero forecast time. The information is exactly one here because the unconditional PDF is symmetric about zero and is divided in half by the conditional PDFs.

**Figure 2.**
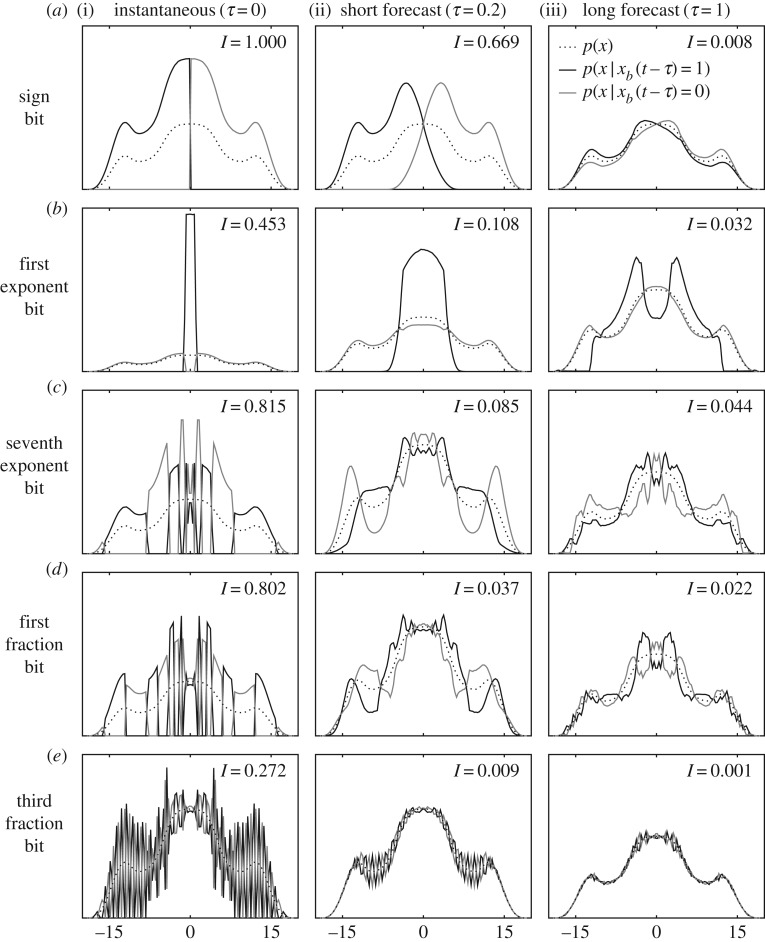
Bit-conditional PDFs and information content for forecasting *x*(*t*+*τ*) in L63. This figure extends the idea in figure 1*d* to five different floating point bits (the sign (*a*), two exponent (*b*,*c*) and two fraction (*d*,*e*) bits), and to three forecast times (current *τ*=0 (i), short *τ*=0.2 (ii) and long *τ*=1 (iii)). In general, the information content (shown in the top right of each panel) decreases for less significant bits of the floating point and as forecast time increases.

Figure 2 applies the information analysis to other bits and forecast times. Starting from figure 2*a*(i) and proceeding to figure 2*a*(iii), we see the decay of information in the sign bit as the forecast time increases. At a relatively short forecast time *τ*=0.2, the sign of *x* usually remains the same, so the conditional PDFs map out mostly non-overlapping regions, thus there is still significant information *I*=0.669 at this forecast time. However, at *τ*=1 the sign bit has lost nearly all of its information content because the sign of *x*(*t*) is nearly impossible to predict one time unit later. Figure 2*b* shows the first bit of the exponent that indicates the sign of the exponent. At zero forecast time, the conditional PDFs cleanly map out whether |*x*|>1. Again the amount of overlapping of the conditional PDFs grows as forecast time extends, and information is lost. Perhaps unexpectedly, the sign of the exponent is found to have more information content than the sign of the number at *τ*=1. This means that knowing whether |*x*|<1 is more important than knowing whether *x*<0 when forecasting 1 mtu into the future.

Figure 2*c*–*e* shows the seventh exponent bit and two of the more significant fraction bits, respectively. The seventh exponent bit is the second least significant bit of the exponent. This means it is true (one) for 2^−3^≤*x*<2^−2^, false (zero) for 2^−2^≤*x*<2^2^, true for 2^2^≤*x*<2^3^, false for 2^3^≤*x*<2^4^, etc., as seen in the *τ*=0 panel (figure 2*c*(i),*d*(i),*e*(i)). The conditional PDFs overlap near zero because of the *h*=0.3 uncertainty. This uncertainty grows and the information content correspondingly decreases with an increase in forecast time. Figure 2*d*,*e* shows the regions of the PDFs mapped out by the first and third most significant fraction bits. The bounds of the regions mapped by these bits are more complicated and distract from our analysis, so we do not explain them here. The general trend is that the less significant the fraction bit, the finer-grained mapping of the number line (finer precision), and the information content for finer-grained mappings decreases faster in forecast time.

**Figure 3.**
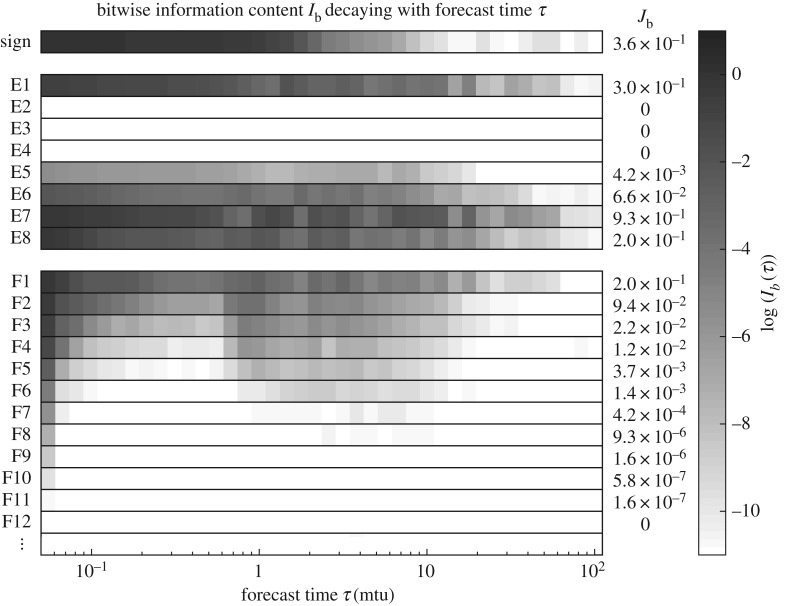
The decay of information content in all of the floating point bits representing *x*(*t*) at forecast time *τ*. The log (base 10) of the information *I*_*b*_(*τ*) for each bit of the sign (*S*), exponent (*E*_*i*_) and fraction (*F*_*i*_) decays as a function of *τ*. Integrating over *τ* gives the total predictive information content *J* for each bit in the floating point representation of *x*(*t*).

Figure 3 shows the log of information content of all bits (except some of the fraction) and at all useful forecast times *I*_*b*_(*τ*) along with the total information content *J*_*b*_. Of the 32 bits used for the floating point type, it is clear that only 16 bits—the sign bit, five of the exponent bits and 10 of the fraction bits—have significant information content. Surprisingly, we find that the bit with the highest total information content is the second least significant bit of the exponent, *J*_*E*7_=0.93. This means that the conditional PDF mapping of this bit (figure 2*c*(i)) is the most helpful for predictability purposes. The most significant exponent bits, E4, E3 and E2, have no information content. Of these bits, the one with the smallest dynamic range is bit E4, which is low for the entire range 2^−5^=0.031≤*x*<2^5^=32. To explain these results, note that no information is contained by mapping out this range because values less than 0.031 are blurred by the uncertainty and values greater than 32 are never reached by the variable in the model. In contrast to the exponent bits, the more significant fraction bits contain more information. Instead of indicating larger and smaller dynamic ranges, fraction bits indicate coarser and finer-grained mappings of the number line. Fraction bits F1 to F10 produce mappings that are more coarse than the higher-order fraction bits, so they provide more useful predictive information content. The fact that *I*_*b*_ is not monotonically decreasing in time for these bits is due to the quasi-periodic nature of the attractor.

The total bitwise information metric *J*_*b*_ in figure 3 is not highly sensitive to decreasing the time step of the model. For example, using a smaller time step might produce a very small amount of information in the fraction bits less significant than F11. However, their total information content *J*_*b*_ would not be significantly changed because this quantity is an integration over the entire useful forecast period. By contrast, *J*_*b*_ is highly sensitive to the uncertainty *h* as slightly smaller (larger) uncertainty in the initial conditions of a chaotic system will significantly improve (diminish) the predictability, thus lengthening (shortening) the useful forecast period.

### (c) L63 testing the analysis

To test our information content metrics, we re-ran the L63 model in three versions of reduced precision: 12 bit, 14 bit and 16 bit. In each version, we retained the sign bit and five bits of the exponent, but varied the number of bits in the fraction. In agreement with the precision analysis, the five exponent bits were found to be mandatory as using anything less resulted in model failure or unrecognizable output. Decreasing the number of fraction bits from 10 to 6, however, showed a slower degradation of quality. The bit limitation was enforced by manually setting the unused bits to zero, using MATLAB’s bitset() command, at the beginning of each time step.^1^ Each reduced precision model was started from the beginning of each 30 mtu forecast segment of the full precision perturbation ***x***′(*t*). We then analysed whether the reduced precision versions are creating a different climatology or faster forecast departure from ***x***(*t*).

**Figure 4.**
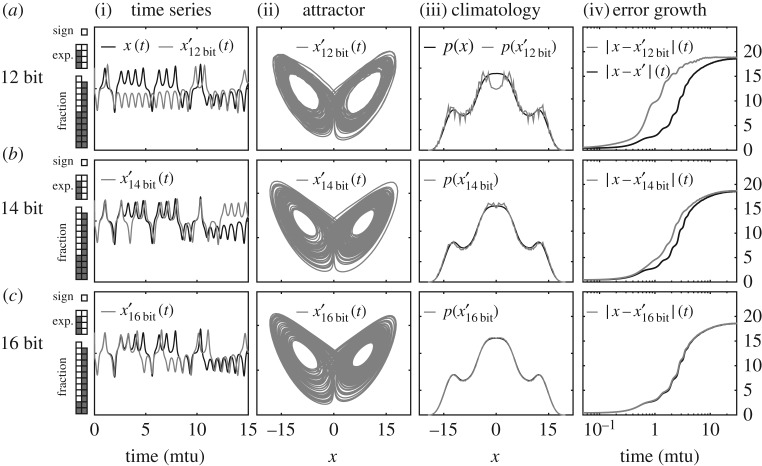
Reduced precision runs of L63 to test the information quantification. The time series (i), attractor (ii), climatology (iii) and error growth rates (iv) are shown for numerically integrating the L63 model in 12-bit (*a*), 14-bit (*b*) and 16-bit (*c*) precision. The graphic on the left indicates which bit is removed (in grey) for each reduced precision run. It is seen that 16-bit precision is satisfactory, given the initial condition uncertainty of *h*=0.3.

The results are shown in figure 4. The schematic on the left indicates which bits were set to zero (coloured grey) in the three reduced precision versions. In figure 4*a*(i)–*d*(i), we see that all of the reduced precision runs (grey) separate from the reference run (black) quickly, but the 16-bit model separates at approximately 4 mtu, the same time as the full precision perturbation *x*′(*t*) in figure 1. The 12- and 14-bit runs also produce noticeably different attractor phase spaces and climatologies, as shown in figure 4*a*(ii,iii)–*d*(ii,iii), whereas the 16-bit model is negligibly different from the full-precision perturbation. Figure 4*a*(iv)–*d*(iv) shows the growth of perturbations averaged over approximately 10^7^ initial conditions. The wavy pattern in the error growth curve is due to the quasi-periodic structure of the attractor, which is independent of the precision reduction, as it is also seen in the full-precision perturbation growth (black line). Thus, the overall result is that, given the initial condition uncertainty of *h*=0.3, we see a negligible difference between a 32-bit and a 16-bit floating point implementation, as predicted by the information content metric.

## 4. Hardware efficiency in the Lorenz 1996 model

In the previous section, we demonstrated the calculation of the bitwise information metric in the L63 model. We now explore how this information can be used to improve forecast accuracy and reduce computational energy consumption in the Lorenz 1996 (L96) model. First, we apply the information metric to the L96 model in the same manner as performed for L63. The results of the analysis for L96 are quite similar to those for L63, so we do not reproduce those figures. Instead we use the analysis to guide the implementation of reduced precision L96 models in an FPGA hardware test bed. We then assess the trade-off of dedicating the finite computational resources of the FPGA to spatial resolution rather than numeric precision in the L96 model. What is shown is that a tolerable reduction in precision, yet major reduction in arithmetic circuitry and energy consumption, occurs when switching from a 32-bit floating point to a 16-bit scaled integer arithmetic.

### (a) L96 experimental set-up

**Figure 5.**
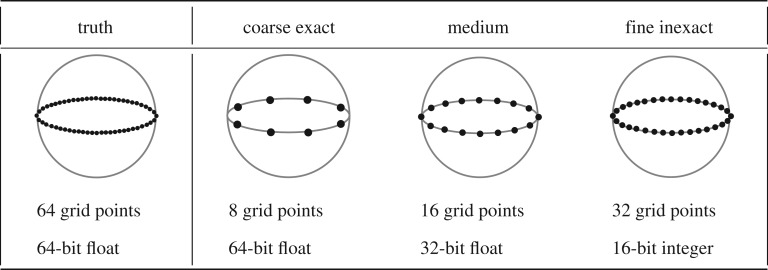
Four versions of the L96 model of atmospheric dynamics along a single latitudinal band. The truth model has the highest spatial resolution and numeric precision. The coarse exact, medium and fine inexact models are implemented on an FPGA and evaluated against the truth model in order to test the trade-off between spatial resolution and numeric precision in terms of energy efficiency and forecast accuracy.

The L96 model was designed to illustrate the chaotic dynamics of atmospheric variables along a single latitudinal band [18]. The model is defined by a system of *K* ordinary differential equations:

4.1 $$\frac{dX_{k}}{dt}=-X_{k-2}X_{k-1}+X_{k-1}X_{k+1}-X_{k}+F,$$

where *k*=1,2,3,…,*K* represent the grid points along the latitudinal band, and the forcing is held constant at *F*=20. As illustrated in figure 5, we construct four versions of this model. First, we construct a truth model (sometimes called a perfect model) that has the highest spatial resolution (*K*=64 grid points) and the highest numeric precision (64-bit floating point). The truth model is implemented only in MATLAB. We then implement three test models that explore the trade-off between resolution and precision in terms of forecast accuracy and computational resources on an FPGA. The coarse exact model has low spatial resolution and high numeric precision (*K*=8- and 64-bit float); the fine inexact model has high spatial resolution and low precision (*K*=32 and 16-bit scaled integer); and the medium model has mid-range values of both (*K*=16 and 32-bit float). There is no real sense of physical space in the equations (e.g. no *Δx*), so we modify the time step in proportion to the resolution (Δ*t*=0.032/*K*) to allow the same speed of signal propagation around the latitudinal band.

The least precise number format used in the test models is the 16-bit scaled integer in the fine inexact model. This level of precision was determined by the bitwise information analysis of the coarse exact model. The analysis looks very similar to that in figure 3 for the L63 model in that only 10 bits of the fraction contained significant information content. This is not surprising as the dynamic range of the L96 variables is approximately −25 to 25 and they experience similar properties of nonlinearity to the *x* variable in L63. Reducing a floating point to 10 fraction bits corresponds to a precision of about four digits after the decimal. This turns out to be approximately the same as a 16-bit integer that uses one bit for the sign, five bits for the integer and 10 bits for the decimal. For example, *π* is 3.1416016 in the 16-bit floating point, and 3.1416015625 in the 16-bit scaled integer. Thus, using the bitwise information analysis, we knew that using a 16-bit integer number format was probably sufficient precision, and this switch would allow more computation to be dedicated to spatial resolution on the FPGA.

The three test models are constructed on an FPGA using tools from Maxeler Technologies.^2^ For each model, all of the arithmetic operations required for one RK4 time step are fully parallelized and laid out in space so that each individual addition and multiplication has its own dedicated circuit. For example, dedicated circuits are constructed for each of the 80 double precision multiplications and 152 double precision additions required for a coarse exact RK4 model time step. The FPGA has a finite amount of logical elements available for circuit construction, so we can compare the percentage of logic utilization for each model. It turns out that the coarse exact model with eight grid points barely fits on the FPGA. A 16-grid point, double precision model would not fit. The medium model has 16 grid points but uses a 32-bit instead of a 64-bit floating point so that its 160 multiplications and 304 additions will fit. The 32 grid points of the fine inexact model using 16-bit scaled integer arithmetic requires 320 multiplications and 608 additions. Special care has to be taken when ordering the operations of the fine inexact model in order to keep the intermediate values of every calculation within the dynamic range of the scaled integer. However, this arithmetic easily fits on the FPGA because the 16-bit integer arithmetic requires approximately 24 times less logic than the 64-bit floating point.

To compare forecast accuracy, we integrate the truth and three test models for 10^6^ mtu with a forecast period of 1 mtu. At the beginning of each forecast period, we reset all models to the most similar state possible, given the different resolutions. We do this by interpolating each resolution to the next highest resolution and adding random values resembling the L96 climatology to the interpolated points. For example, at the beginning of each forecast period the values on grid points 1, 3, 5, 7, 9, 11, 13, 15 of the medium model are set equivalent to grid points 1, 2, 3, 4, 5, 6, 7, 8 of the exact coarse model, and random values are assigned to points 2, 4, 6, 8, 10, 12, 14, 16 of the medium model. The random values are drawn from a Gaussian approximation of the L96 climatology (mean 3.6 and s.d. 4). The same interpolation procedure is applied from the medium to the coarse exact model, and from the coarse exact model to the truth model. Applying this procedure results in each model being identical to each other model at the wavenumbers shared by the models (at the beginning of each forecast period). As the forecast period extends, the models drift away from one another and the forecast error is measured only on the lowest wavenumber signal (*K*=8) of all models. Thus, the forecast error measures how errors in the low wavenumber signal grow due to higher wavenumber signals that are not representable in the coarser models.

### (b) L96 efficiency and accuracy results

**Figure 6.**
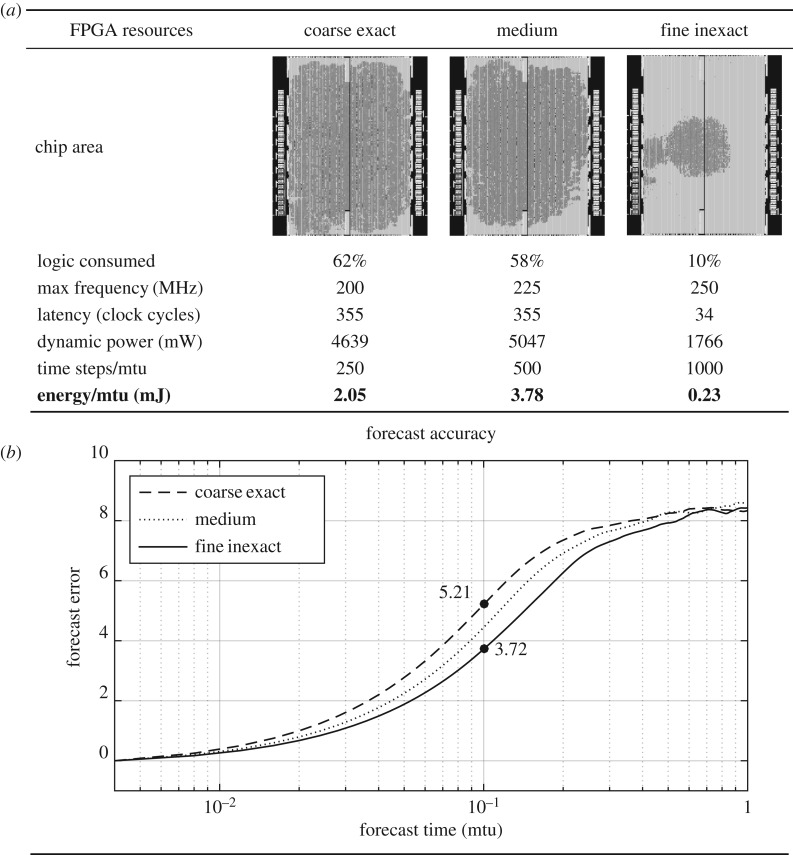
FPGA resources (*a*) and forecast accuracy (*b*) of the three test models. FPGA designs were created on a Maxeler Data Flow Engine, which uses an Altera Stratix V FPGA. Altera’s Quartus II software provided the chip area and resource report. Energy/mtu is calculated via equation (4.2) in the main text. Even though the fine inexact model has four times more spatial resolution, it consumes approximately 10 times less computational resources and energy than the coarse exact model because of the simplicity of the integer arithmetic compared with the floating point. In the forecast accuracy plot at 0.1 mtu, the fine inexact model’s forecasts are 28.6% better than those of the coarse exact model because the error in numeric precision is negligible compared with the accuracy gain from increased spatial resolution.

Figure 6 displays the FPGA resources and forecast accuracy of the three test models. The chip area and resource report where generated by the Quartus II software provided the FPGA manufacturer, Altera. Overall, the fine inexact model requires approximately 10-fold less computing resources than the coarse exact and medium models. The dark grey regions in the chip area images indicate areas of the chip where a significant amount of the FPGA’s available logic was consumed. The coarse exact and medium models both consume nearly around 60% of the logic. This similarity is because the higher precision of the coarse exact model trades off equally with the higher resolution of the medium model. Double precision arithmetic on eight grid points requires almost the same resources as single precision arithmetic on 16 grid points. The fine inexact model makes a significantly smaller footprint on the chip as it consumes only 10% of the logic. Owing to this smaller footprint, there is less circuit propagation delay, so the fine inexact model is able to operate at a faster speed (250 MHz) than the other two models (200 and 225 MHz). The simplicity of integer arithmetic is seen not only in the amount of logic, but also in the number of clock cycles needed for each arithmetic operation. For example, a double precision floating point addition requires 14 clock cycles, whereas a 16-bit integer requires only one. And because not all of the time-step arithmetic can be performed simultaneously, the coarse exact and medium models require 355 clock cycles per time step, whereas the fine inexact model requires only 34. The dynamic power of the models, also provided by the Quartus II resource report, is an estimate of the power consumed while bits are actively propagating through the device. While still much greater, the fine inexact model consumes approximately only three times less dynamic power because a basic amount of dynamic power is needed even for the simplest logic. To calculate the total amount of energy consumed *E* (joules) by each model, we must take into account not only the dynamic power rate *P* (watts=J s^−1^) but also the total running time. The total running time is calculated using the number of clock cycles *N* needed for a time step, the operating frequency of the device *F* (Hz=1 s^−1^) and the number of equivalent time steps *T* (due to the different size of the time steps for different resolutions):

4.2 $$E=P\times \frac{NT}{F}.$$

The result is that the fine inexact model consumes approximately 10 times less energy than the coarse exact or Medium models.

Figure 6*b* compares the forecast accuracy of the models. The forecast error is the root mean squared error between the test models and the truth model at the *K*=8 wavenumber (explained above) and as a function of forecast time. The error is averaged over 10^5^ forecast periods. For all models, the error begins at zero and stabilizes at its maximum error by 1 mtu. At 0.1 mtu, the fine inexact model shows a 28.6% improvement in forecast accuracy compared with the coarse exact model. This shows that the error in numeric precision in the fine inexact model is negligible compared with the accuracy it has gained from its increased spatial resolution.

## 5. Conclusion

In this study, we have introduced a bitwise information metric for chaotic models, and demonstrated how removing bits with negligible information content reduces the energy required to computationally integrate the model. The metric quantifies the information content of bits in relation to the growth of an uncertainty in the chaotic system. We applied the metric to the well-known L63 model with an initial condition uncertainty of *h*=0.3 and found that only 16 of the 32-bit number format contained significant information. We then re-ran the model, in a reduced precision mode, and verified that using only 16 bits indeed did not add any additional error on top of the growth of the *h*=0.3 initial condition uncertainty. This result is specific to the level of uncertainty and model parameters, but the metric we have introduced can be used to identify the information content of bits for any non-zero uncertainty or choice of parameters in a chaotic model.

The second part of the study demonstrated a significant gain in energy efficiency at the hardware level of an FPGA, by removing the low information content bits and increasing the spatial resolution of the L96 model. In the L96 model, uncertainty arises from the unresolved dynamics of sub-grid-scale processes. Given the finite amount of bits available in an FPGA, a trade-off must be made between the number of bits used to represent a variable at a grid location and the number of grid locations that contain variables. We were guided in this trade-off by using the bitwise information metric to identify bits that contained negligible information (compared with the growth of the sub-grid-scale errors), and then repurposed those bits for increased spatial resolution. More specifically, we found that a 16-bit integer format would suffice for the L96 model, given our choices of parameters. We then tested the trade-off between numeric precision and spatial resolution by constructing three hardware-level implementations: a coarse exact model (low spatial resolution, high numeric precision), a medium model and a fine inexact model (high spatial resolution, low numeric precision). The result was that the fine inexact model was both more accurate (up to 28% better forecasts) and more energy efficient (six times fewer logic operations and 10 times less power, per model time step) than the coarse exact model. The efficiency gains were largely due to the simplicity of the integer over floating point arithmetic at the hardware level. Similar to the first part of this study, the efficiency gains of the L96 FPGA implementations are specific to the model parameters chosen. However, we suggest that the process of identifying and then removing bits of low information content, as demonstrated in this paper, can be used to the improve the energy efficiency of integrating a large class of chaotic models in the presence of uncertainty.

## Footnotes

1 In MATLAB R2015a the command bitset() only works on integers, so we typecast() the float to an integer (preserving the bit pattern), set the bits and then typecast back to float.

2 We use Maxeler’s MAIA MAX4848A Desktop Data Flow engine, which operates on a Altera Stratix V FPGA.

## Data accessibility

The code and data are freely available at https://github.com/StephenJeffress/BitwiseInfo.

## Appendix A. Computational solution

The L63 model is integrated numerically using the MATLAB development environment. After discarding the transient behaviour associated with a random initial condition, the model is integrated for 10^8^ mtu with an RK4 method, and a time step of Δ*t*=0.01 mtu. The model parameters are set to the values most commonly used in studies of the system, *σ*=10, $\beta =\frac{8}{3}$ and *ρ*=28.

The three L96 test models are constructed on an FPGA using tools from Maxeler Technologies. We use Maxeler’s MAIA MAX4848A Desktop Data Flow engine, which operates on an Altera Stratix V FPGA. For each model, all of the arithmetic operations required for one RK4 time step are fully parallelized and laid out in space so that each individual addition and multiplication has its own dedicated circuit.
